# A long-term, phase 2, multicenter, randomized, open-label, comparative safety study of pomaglumetad methionil (LY2140023 monohydrate) versus atypical antipsychotic standard of care in patients with schizophrenia

**DOI:** 10.1186/1471-244X-13-143

**Published:** 2013-05-22

**Authors:** David H Adams, Bruce J Kinon, Simin Baygani, Brian A Millen, Isabella Velona, Sara Kollack-Walker, David P Walling

**Affiliations:** 1 Eli Lilly and Company, Indianapolis, IN 46285, USA; 2 Collaborative NeuroScience Network, Inc, Garden Grove, CA 92845, USA

**Keywords:** Schizophrenia, Glutamate, Olanzapine, Metabotropic glutamate receptor 2 (mGluR2), Metabotropic glutamate receptor 3 (mGluR3)

## Abstract

**Background:**

We compared the time to discontinuation due to lack of tolerability over 24 weeks in patients suffering from schizophrenia treated with pomaglumetad methionil (LY2140023 monohydrate, the prodrug of metabotropic glutamate 2/3 receptor agonist, LY404039) or standard of care (SOC: olanzapine, risperidone, or aripiprazole).

**Methods:**

Study HBBR was a multicenter, randomized, open-label study comparing the long-term safety and tolerability of LY2140023 with SOC for schizophrenia. Patients had moderate symptomatology with prominent negative symptoms and evidence of functional impairment. Those who met entry criteria were randomized to open-label treatment with either LY2140023 (target dose: 40 mg twice daily [BID]; n = 130) or SOC (n = 131).

**Results:**

There was no statistically significant difference between LY2140023 and SOC for time to discontinuation due to lack of tolerability (primary objective; P = .184). The Kaplan-Meier estimates revealed comparable time to event profiles. Only 27% of LY2140023 and 45% of SOC patients completed the 24-week open-label, active treatment phase. Twenty-seven patients (20.8%) in the LY2140023 group and 15 patients (11.5%) in the SOC group discontinued due to lack of efficacy (P = .044). Twenty-three patients (17.7%) in the LY2140023 group and 19 patients (14.5%) in the SOC group discontinued due to adverse events (physician and subject decision combined, P = .505). The incidence of serious adverse events was comparable between groups. LY2140023-treated patients reported significantly more treatment-emergent adverse events of vomiting, agitation, and dyspepsia, while SOC-treated patients reported significantly more akathisia and weight gain. The incidence of treatment-emergent parkinsonism (P = .011) and akathisia (P = .029) was significantly greater in SOC group. Improvement in PANSS total score over the initial 6 to 8 weeks of treatment was similar between groups, but improvement was significantly greater in the SOC group at 24-week endpoint (P = .004). LY2140023 and SOC groups had comparable negative symptom improvement at 24-week endpoint (P = .444).

**Conclusion:**

These data provide further evidence that the potential antipsychotic LY2140023 monohydrate, with a glutamatergic mechanism of action, may have a unique tolerability profile characterized by a low association with some adverse events such as extrapyramidal symptoms and weight gain that may characterize currently available dopaminergic antipsychotics.

**Trials registration:**

A Long-term, Phase 2, Multicenter, Randomized, Open-label, Comparative Safety Study of LY2140023 Versus Atypical Antipsychotic Standard of Care in Patients with DSM-IV-TR Schizophrenia

ClinicalTrials.gov identifier: NCT00845026.

## Background

LY2140023 monohydrate (LY2140023) is an oral methionine prodrug of LY404039, a potent and highly selective agonist for the metabotropic glutamate receptors 2 and 3 (mGlu2/3) [1,2]. LY2140023 is undergoing testing in clinical trials as a potential treatment for schizophrenia. In a randomized, double-blind, placebo- and active-controlled (olanzapine) clinical trial (HBBD) over a 4-week treatment period, patients receiving 40 mg twice daily (BID) of LY2140023 demonstrated statistically significant improvements in the Positive and Negative Syndrome Scale (PANSS) [3] total score compared with patients receiving placebo [4].

The efficacy results in a second, randomized, double-blind, placebo- and active-controlled (olanzapine) clinical trial (HBBI) lasting up to 4 weeks were inconclusive [5]. None of the 4 LY2140023 doses (5 mg, 20 mg, 40 mg, or 80 mg BID) nor the olanzapine dose (15 mg once daily) demonstrated changes different from placebo in the PANSS total score, the primary measure of efficacy. The heightened placebo response in the second phase 2 study likely reduced the ability to detect a significant drug response across the LY2140023 and olanzapine treatment groups.

The safety results from both 4-week, Phase 2 clinical trials comparing LY2140023 with placebo treatment were generally similar. LY2140023 treatment was generally well-tolerated overall and not associated with elevations in prolactin, extrapyramidal symptoms, or weight. However, in the HBBI study, convulsions were reported in 4 patients treated with LY2140023 [5]. This latter finding identified a potentially increased risk of seizures during treatment with LY2140023, but further study is needed to evaluate any potential relationship to duration of exposure.

The current study (HBBR) is the first time patients were assigned to long-term treatment with LY2140023. It is important for assessing any potential association between prolonged duration of exposure to LY2140023 and the incidence of convulsions. The primary objective of this study was to assess time to discontinuation due to lack of tolerability among patients with schizophrenia receiving LY2140023 versus those on atypical antipsychotic standard of care (SOC) treatment for up to 24 weeks.

## Methods

### Study design

**Figure 1:**
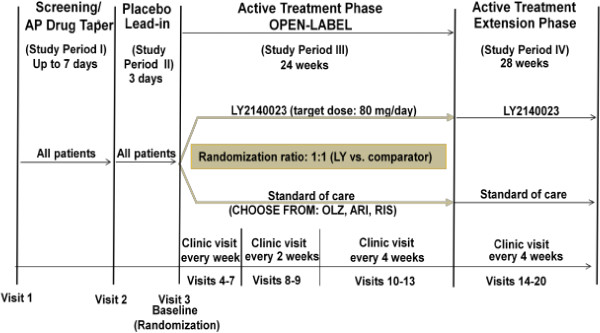
**Study design.** Abbreviations: AP = antipsychotic; ARI = aripiprazole; OLZ = olanzapine; LY = Lilly2140023; RIS = risperidone; SOC = standard of care; vs. = versus. Dose range: LY2140023: 20, 40, or 80 mg, twice daily; ARI: 10, 20, or 30 mg, once daily; OLZ: 10, 15, or 20 mg, once daily; RIS: 2, 4, or 6 mg, once or twice daily.

Study HBBR was a Phase 2, multicenter, randomized, parallel, active-controlled study with an open-label design (Figure 1). Patients enrolled in this study were male or female, 18 to 65 years of age (inclusive), with a diagnosis of schizophrenia as defined in the Diagnostic and Statistical Manual of Mental Disorders – Fourth Edition – text revision (DSM-IV-TR) [6]. Diagnosis was confirmed with the Structured Clinical Interview for Diagnosis (SCID), performed by the principal investigator. Patients had at most moderate symptomatology, as reflected by a score ≤4 (moderate) on the Clinical Global Impressions-Severity (CGI-S) scale [7], with prominent negative symptoms defined as symptom score ≥4 on at least 3 items or a score ≥5 on at least 2 items of the PANSS negative subscale [3], and evidence of functional impairment at study entry. Assessment of “functional impairment” was based on the clinical judgment of the principal investigator. All investigators were trained that functional impairment was a deficiency in the expected tasks of patients as appropriate for a student, homemaker, or worker. Patients were not eligible to participate in the study if they had a history of seizures or additional specified criteria that indicated seizure tendency. All patients gave written informed consent prior to entering the study. The study protocol was approved by appropriate institutional review boards and conducted in accordance with the ethical principles stated in the Declaration of Helsinki. This study was conducted from 2009 through 2010 at 29 centers in 4 countries; the number of patients in each country were: Germany (n = 10, 3.8%), Mexico (n = 48, 18.4%), Russia (n = 86, 33.0%), and the United States (n = 117, 44.8%).

The study included an up to 7-day antipsychotic drug taper/discontinuation phase (Study Period I), a 3-day, single-blind placebo lead-in phase (Study Period II), a 24-week open-label active treatment phase (Study Period III), and an optional 28-week extension phase (Study Period IV). All results pertain to Study Periods I through III only. Patients were randomized to receive either LY2140023 (20, 40, or 80 mg, BID; target dose = 40 mg BID), or SOC (olanzapine [10, 15, or 20 mg, once daily; target = 15 mg/day], aripiprazole [10, 20, or 30 mg, once daily; target = 20 mg/day], or risperidone [2, 4, or 6 mg, once or twice daily; target = 4 mg/day]) treatment. Doses of comparators were based on U.S. labeling. Based upon the available Phase 2 data, an optimal dose of LY2140023 had not yet been identified.

The treating clinician was free to select which SOC antipsychotic to use for each patient randomly assigned to SOC treatment; the clinician was not permitted to switch a patient to another SOC medication once treatment began. Patients randomized to receive LY2140023 were started at a dose of 40 mg BID. For patients randomized to receive SOC treatment, investigators were instructed to begin patients at doses below the target dose for the first 3 days to minimize the potential for adverse events. Dose adjustments, other than the initial scheduled dose titration, were made only if investigators had concerns about patient safety/tolerability or lack of response. Dose decreases to accommodate investigator concern about a patient’s safety or tolerability to study medication could occur at any time, based on the investigator’s clinical judgment; only 1 dose decrease was permitted between visits. Dose decreases for patients receiving LY2140023 should be from 80 mg/day to 40 mg/day (with no intermediate dose). Dose increases to potentially improve clinical response could occur no earlier than Visit 6 (Week 3) to ensure sufficient time for patients to respond, and were made only at scheduled visits. Investigators were permitted to increase the dose for LY2140023 patients to 160 mg/day (80 mg BID), with no intermediate dose. Once patients reached the target dose, dose adjustments for patients on comparator medications could occur in the following increments: olanzapine (+/− 5 mg/day), aripiprazole (+/− 10 mg/day), or risperidone (+/− 2 mg/day).

### Assessments

The primary objective of this study was to assess time to discontinuation due to lack of tolerability, defined as discontinuation due to adverse events, among patients with schizophrenia receiving LY2140023 versus those on atypical antipsychotic SOC treatment for up to 24 weeks. The classification of discontinuation due to adverse events was based on the site investigator’s determination that the primary reason for a patient's early withdrawal from the study was an adverse event.

The secondary objectives of this study included evaluation of the long-term safety, tolerability, and efficacy of LY2140023 compared with SOC treatment. Safety assessments included the incidence of treatment-emergent adverse events (TEAEs), neurological changes, vital signs, laboratory changes, and changes in electrocardiographs and electroencephalographs (EEGs). Extrapyramidal symptoms were assessed by the Barnes Akathisia Scale for akathisia [8], Simpson-Angus Scale for parkinsonism [9], and Abnormal Involuntary Movement Scale for dyskinetic symptoms [10].

All patients had an EEG at baseline and at Weeks 2, 4, 8, 12, and 24 that were evaluated by a single central neurologist. All EEGs were performed on the same model device utilizing the same software and settings.

Efficacy was assessed by the PANSS [3], CGI-S [6], and the 16-item Negative Symptoms Assessment (NSA-16) scale [11]. Raters not directly involved with the patient’s treatment were used to assess the PANSS and NSA-16 scales. The raters were blind to the study design, entrance criteria, and treatment assignment; separate raters were used for baseline and endpoint assessments.

### Statistical methods

All analyses were conducted on an intent-to-treat basis, meaning that data were analyzed by the treatment groups to which patients were randomly assigned, even if the patient did not take the assigned treatment, did not receive the correct treatment, or did not comply with the protocol. No adjustments were made for multiplicity. All treatment comparisons were evaluated based on a 2-sided significance level of 0.05.

Unless otherwise specified, when an analysis of variance (ANOVA) model or an analysis of covariance (ANCOVA) model was used to analyze a continuous efficacy variable, the model contained the main effects of treatment and investigator. Type III sum of squares for the least-squares (LS) means were used for statistical comparison from ANOVA or ANCOVA models.

For the analyses of change from baseline, only patients with a baseline and at least 1 postbaseline measure were included. Unless otherwise specified, baseline was defined as the last nonmissing observation at or before the randomization visit (Visit 3). Endpoint for Study Period III (24 weeks) was defined as the last nonmissing observation obtained from Visit 4 through Visit 13.

The primary safety outcome was the time to discontinuation due to adverse events during Study Period III. Kaplan-Meier estimated survival curves [12] of time to discontinuation (measured in days) for adverse events were compared between LY2140023 and SOC. The log-rank test was used to compare the LY2140023 and SOC time-to-discontinuation profiles. This study was planned to provide approximately 80% power to detect a hazard ratio of 0.36 in the time to discontinuation due to adverse events over the 24-week active treatment phase of the study.

Secondary efficacy outcomes for the PANSS total and subscores were analyzed using the mixed-model repeated measures (analysis) model (MMRM), with the fixed categorical effects of treatment, gender, investigator, visit, and treatment-by-visit interaction, as well as the continuous, fixed covariate of baseline score and baseline-by-visit interaction.

## Results

### Baseline and demographic characteristics

Patients in the 2 groups did not significantly differ with respect to baseline characteristics (Table 1). More than half of patients were male (65.9%) and white (52.1%). Mean age was approximately 39 years (standard deviation [SD] = 11.7). The mean PANSS total score was 85.38 (SD = 15.26), reflecting moderate overall symptomatology, and the mean PANSS negative score was greater than the PANSS positive score, reflecting prominent negative symptoms, as specified in the protocol.

**Table 1:** Baseline and demographic characteristics

**Characteristic**	**LY2140023**	**SOC**	*p *Value*
	**(N = 130)**	**(N = 131)**	
Age, years, mean (SD)	38.7 (10.9)	39.5 (12.5)	.579
Gender – female, n (%)	45 (34.6)	44 (33.6)	.897
Race, n (%)	-	-	.669
American Indian or Alaska Native	20 (15.4)	18 (13.7)	-
Black/African American	44 (33.8)	39 (29.8)	-
Multiple	1 (0.8)	3 (2.3)	
White	65 (50.0)	71 (54.2)	-
Diagnosis, n (%)	-	-	.202
Catatonic	1 (0.8)	0 (0.0)	-
Disorganized	8 (6.2)	4 (3.1)	-
Paranoid	100 (76.9)	109 (83.2)	-
Residual	7 (5.4)	2 (1.5)	
Undifferentiated	14 (10.8)	16 (12.2)	-
Weight, kg, mean (SD)	83.0 (21.5)	82.7 (21.7)	.907
Age of first episode, years, mean (SD)	24.6 (8.3)	23.1 (8.5)	.148
Episodes in the last 24 months, mean (SD)	1.8 (1.6)	2.0 (2.1)	.297
CGI-S, mean (SD)	3.8 (0.7)	3.7 (0.6)	.943
PANSS total, mean (SD)	85.6 (14.8)	85.1 (15.8)	.788
PANSS positive, mean (SD)	17.6 (4.7)	18.1 (4.8)	.355
PANSS negative, mean (SD)	26.2 (4.9)	25.7 (5.2)	.403
PANSS general psychopathology, mean (SD)	41.9 (9.1)	41.4 (9.3)	.644
Negative Assessment Scale-16, mean (SD)	58.7 (11.0)	57.3 (11.3)	.336

Abbreviations: *CGI-S* Clinical global impressions Severity, *n/N* Number of patients, *PANSS* Positive and negative syndrome scale, *SD* Standard deviation.

Groups: *LY2140023*, LY2140023 monohydrate, *SOC*, Standard-of-care.

^*^P < .05, LY2140023 versus SOC.

### Dosing

The majority of LY2140023-treated patients were taking the targeted dose (40 mg BID) at their final visit of the Study Period (approximate incidence of patients in each dose group at final visit: 14% patients at 20 mg BID; 62% patients at 40 mg BID; and 25% patients at 80 mg BID). The percentage of patients in the SOC group at their final visit of the Study Period receiving the minimum dose was 17%, the targeted dose was 46%, and the percentage receiving maximum dose was 38%. For each drug subgroup within the SOC group, the percentage of patients at their final visit of the study receiving the minimum, targeted, and maximum doses were: OLZ, 20%, 36%, 44%; RIS, 16%, 60%, 24%; and ARI: 14%, 48%, 39%, respectively.

### Patient disposition

A total of 402 patients entered the study, and 261 patients were randomized to BID doses of LY2140023 (n = 130 patients) or SOC treatment (n = 131 patients); 27% of LY2140023 and 45% of SOC patients completed through the final visit of Study Period III. The most common reasons for study discontinuation among patients in both treatment groups were perceived lack of efficacy, adverse events, and withdrawn consent. Twenty-seven patients (20.8%) in the LY2140023 group and 15 patients (11.5%) in the SOC group discontinued due to lack of efficacy (physician and subject decision combined; P = .044). Twenty-three patients (17.7%) in the LY2140023 group and 19 patients (14.5%) in the SOC group discontinued due to adverse events (physician and subject decision combined, P = .505).

### Primary objective – time to discontinuation due to adverse events

**Figure 2:**
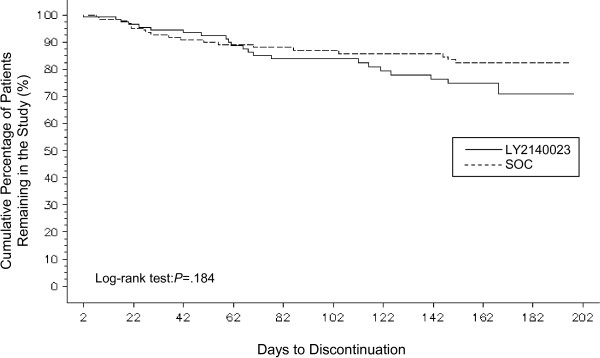
**Kaplan-Meier time to discontinuation due to adverse events.** There was no statistically significant difference between the LY2140023 and SOC groups in time to discontinuation due to lack of tolerability through 24 weeks (log-rank test; P = .184).

There was no statistically significant difference between the LY2140023 and SOC groups in time to discontinuation due to lack of tolerability through 24 weeks (log-rank test; P = .184; Figure 2).

However, time to discontinuation for any reason (p = .002) was significantly shorter for patients in the LY2140023 group than for those on SOC (data not shown).

### Other safety measures

A total of 30 patients (LY2140023, n = 18; SOC, n = 12) experienced at least 1 serious adverse event (SAE) during the 24-week active-treatment phase of the study. The incidence of SAEs was not significantly different between groups. Reported SAEs included 3 deaths: 2 were in patients randomized to LY2140023, and 1 was in a patient who had not yet received any study drug. None of the 3 deaths was considered by investigators to be related to treatment with study drug or to protocol procedures.

One patient experienced 2 convulsions 1 day after discontinuing LY2140023 (approximately 3 weeks after randomization) and initiating treatment with intramuscular haloperidol; the convulsions were reported as possibly related to LY2140023 treatment. Another patient experienced a convulsion during the placebo lead-in phase; this convulsion was reported as possibly related to protocol procedure.

SAEs commonly reported (≥1% of LY2140023 patients) in the LY2140023 group were schizophrenia, psychotic disorder, agitation, insomnia, and pneumonia. Among patients in the SOC group, common SAEs were schizophrenia and psychotic disorder.

TEAEs reported at a statistically significantly higher rate among LY2140023-treated patients than among those on SOC included vomiting (P = .003), dyspepsia (P = .019), and agitation (P = .011) (Table 2). Among patients in the SOC group, akathisia (P = .019) and weight gain (P = .007) were reported at a significantly greater rate compared with the rate among those in the LY2140023 group.

**Table 2:** Treatment-emergent adverse events at incidence ≥5% in the LY2140023 group, or significantly different between groups

**Event, n (%)**	**LY2140023**	**SOC**	**P value**
	**(N = 130)**	**(N = 131)**	
Patients with ≥ TEAE	100 (76.9)	94 (71.8)	.396
Insomnia	20 (15.4)	10 (7.6)	.054
Anxiety	19 (14.6)	10 (7.6)	.079
Headache	16 (12.3)	10 (7.6)	.222
**Vomiting**	**15 (11.5)**	**3 (2.3)**	.003*
Schizophrenia	12 (9.2)	11 (8.4)	.831
**Agitation**	**11 (8.5)**	**2 (1.5)**	.011*
Blood CPK increases	10 (7.7)	14 (10.7)	.521
Nausea	10 (7.7)	4 (3.1)	.108
**Dyspepsia**	**8 (6.2)**	**1 (0.8)**	.019*
Nasopharyngitis	7 (5.4)	3 (2.3)	.217
**Akathisia**	**1 (0.8)**	**9 (6.9)**	.019^*^
**Weight increased**	**0 (0.0)**	**8 (6.1)**	.007*

Abbreviations: *CPK* Creatinine phosphokinase, *n/N* Number of patients, *SOC* Standard of care, *TEAE* Treatment-emergent adverse events.

Groups: *LY2140023*, LY2140023 monohydrate, *SOC*, Standard-of-care.

*P < .05, LY2140023 versus SOC. Events significantly different between treatment groups are highlighted in bold text.

Within-group comparisons revealed a significant reduction from baseline to last observation in fasting cholesterol (−0.30 mmol/L, P < .001), LDL cholesterol (−0.28 mmol/L, P < .001), and triglycerides (−0.11 mmol/L, P = .008) in patients treated with LY2140023, and a significant decrease in HDL cholesterol (−0.06 mmol/L, P = .004) in patients treated with SOC. There was a mean decrease in LDL cholesterol in the LY2140023 group that was significantly greater than the decrease observed in the SOC group (P = .031), and a decrease in the level of triglycerides in the LY2140023 group versus an increase in the SOC group (P = .008). The mean decrease (−0.06 mmol/L) in HDL cholesterol observed in the SOC group was significantly different from the slight increase (0.01 mmol/L) observed in the LY2140023 group (P = .018). A significant reduction from baseline was also observed for prolactin (−4.33 μg/L, P = .003) in the LY2140023 group, although prolactin changes did not differ between the groups (P = .062). There were no other clinically significant differences in mean laboratory values between treatment groups.

Within-group comparisons showed significant increases in weight at every visit for patients in the SOC group (maximum increase: 3.12 kg, P < .001), while LY2140023-treated patients had significant decreases in weight at Weeks 12, 16, and 24 (maximum decrease: -2.04 kg, P < .05). Pairwise comparisons revealed significant differences in mean weight change between LY2140023- and SOC-treated patients at every visit. Significantly more patients in the SOC group met potentially clinically significant (PCS) criteria of an increase in weight of at least 7% from baseline to endpoint (SOC, 21.4%, n = 28; LY2140023, 8.2%, n = 10; P = .004), while significantly more patients in the LY2140023 group met PCS criteria for decreased weight (SOC, 2.3%, n = 3; LY2140023, 14.8%, n = 18; P < .001).

Within-group comparisons showed a mean reduction from baseline in the Barnes Akathisia Scale global score for akathisia at most visits in the LY2140023 group (P < .05), while changes from baseline in the SOC group generally did not reach significance. Both treatment groups had statistically significant, within-group improvements on the Simpson-Angus Scale total score for parkinsonism at each visit (P < .001, most visits). There were few statistically significant differences observed from baseline to postbaseline visit in the Abnormal Involuntary Movement Scale total score for dyskinetic symptoms within either treatment group. Pairwise comparisons revealed few statistically significant differences between the LY2140023 and SOC treatment groups across these 3 extrapyramidal symptom measures (data not shown). The overall incidence of treatment-emergent parkinsonism (SOC, n = 14; LY2140023, n = 3; P = .011) and akathisia (SOC, n = 18; LY2140023, n = 6; P = .029) was significantly higher among patients treated with SOC than among patients in the LY2140023 group.

Comparisons of changes in electrocardiogram intervals and heart rate did not show significant differences between groups that were clinically significant.

There were no clinically significant differences in baseline EEG scores between groups and no clinically significant changes in postbaseline EEG scores. Abnormal epileptiform-like changes were not frequent or remarkably different between treatment arms (LY2140023, 0%; SOC, 3.4%).

### Efficacy measures

**Figure 3:**
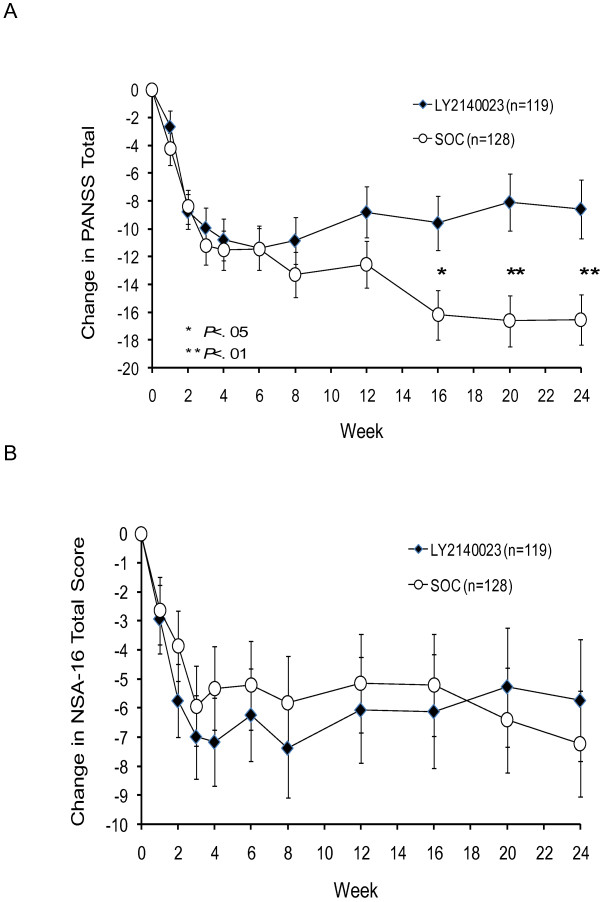
**Efficacy measures. A**. LS mean change from baseline to endpoint in PANSS total score in the LY2140023 and SOC treatment groups estimated from the MMRM model; error bars indicate standard error. Mean PANSS total score at baseline (SD): LY2140023: 85.6 (14.8); SOC: 85.1 (15.8). For the PANSS Total Score, there was a significant effect of baseline (Overall MMRM, F [1,277] = 55.10, P < .001), and a significant effect of treatment (Overall MMRM, F [1,173] = 4.20, P = .042). **B**. LS mean change from baseline to endpoint in NSA-16 total score in the LY2140023 and SOC treatment groups estimated from the MMRM model; error bars indicate standard error. Mean NSA-16 total score at baseline (SD): LY2140023: 58.7(11.0); SOC: 57.3 (11.3). For the NSA-16 Total Score, there was a significant effect of baseline (Overall MMRM, F [1,207] = 36.55, P < .001), but not a significant effect of treatment (Overall MMRM, F [1,180] = 0.39, P = .532). Abbreviations: LS = least squares; MMRM = mixed-model repeated measures; NSA-16 = 16-item Negative Symptoms Assessment scale; PANSS = Positive and Negative Syndrome Scale; vs. = versus. Groups: LY2140023 = LY2140023 monohydrate, SOC = standard-of-care. *P < .05, LY2140023 versus SOC.

Patients in both treatment groups demonstrated a statistically significant within-group mean reduction from baseline in the PANSS total score at each visit (Figure 3A). A similar mean reduction in the PANSS total score was observed for patients in both groups from Weeks 1 to 6, while the improvement in psychopathology was significantly greater in SOC-treated patients at Week 16 (P = .012), Week 20 (P = .002), and Week 24 (P = .004). A similar response pattern as seen for the PANSS total score was generally observed both within and between the 2 treatment groups for the PANSS positive and general psychopathology subscores, and for the CGI-S (data not shown).

Patients in both treatment groups showed a statistically significant within-group mean reduction in the PANSS negative subscore at all visits, with no significant differences observed between the LY2140023 and SOC treatment groups (data not shown). Similarly, patients in both treatment groups showed statistically significant within-group mean improvement in the NSA-16 scale, with no significant between-group differences (Figure 3B).

### Medication compliance

Patient compliance with study medication was assessed at each visit by direct questioning and by counting returned tablets. Patients in both treatment groups showed relatively high compliance rates with no between-group differences (mean percentage study drug compliance: SOC, 88%; LY2140023, 87%; P = .539).

### Concomitant medications

There were no statistically significant differences in the incidence of concomitant use of any class of medication (benzodiazepines/hypnotics/anxiolytics, antidepressants or mood stabilizers, anticholinergic agents, or other therapies) observed between the treatment groups during the active treatment period. Approximately half of patients in this study reported the use of at least 1 concomitant benzodiazepine anxiolytic, or hypnotic agent.

## Discussion

This study was a multicenter, randomized, open-label, phase 2 clinical trial designed to evaluate the long-term safety and tolerability of LY2140023 monohydrate (LY2140023) in moderately ill patients with schizophrenia who had prominent negative symptoms and evidence of functional impairment. The primary analysis showed LY2140023 to be comparable to SOC in time to discontinuation due to lack of tolerability through 24 weeks of treatment. There were no significant differences between treatment groups in the incidence of SAEs, TEAEs, or adverse events leading to study discontinuation. However, a significantly greater number of patients in the LY2140023 group discontinued from the study due to perceived lack of efficacy compared with those in the SOC group. In addition, time to discontinuation for any reason was significantly shorter for patients in the LY2140023 group than for those on SOC. All treatments in the study were generally well-tolerated.

The safety findings from this 24-week study were generally consistent with observations from the 2 previous, 4-week clinical trials. In these acute trials, LY2140023-treated patients generally did not differ significantly from placebo-treated patients with regard to prolactin elevation, extrapyramidal symptoms or weight gain, while comparisons between placebo and olanzapine revealed weight gain and limited or no reduction in prolactin in the olanzapine-treated patients. In the current study, LY2140023 treatment was characterized not only by a decrease in mean weight over 24 weeks, as compared with an increase observed with SOC treatment, but also by improvement across various metabolic parameters. LY2140023 was associated with a reduction in akathisia, and with less treatment-emergent akathisia and parkinsonism as compared with SOC treatment. This unique tolerability profile highlights possible advantages of a glutamate-based schizophrenia therapy versus dopaminergic-based therapies.

The occurrence of convulsions within the 4-week HBBI study identified a potentially increased risk of seizures during treatment with LY2140023, warranting further study following long-term exposure. In the current study, there were 2 patients who experienced convulsive events, 1 patient treated with LY2140023 and a second patient treated with placebo prior to randomization. The overall LY2140023 exposure-adjusted rate of seizures had decreased substantially with the increased compound exposure in the current 24-week HBBR study and in other ongoing studies. Extensive repeat EEG assessments did not demonstrate any abnormal epileptiform-like changes that were frequent nor remarkably different between treatment arms. Study results do not suggest an increased risk of seizures following long-term exposure. Based on results of the current study, the safety profile of LY2140023 appears similar after short- and long-term exposure.

Efficacy was a secondary endpoint in this study. Improvement on the PANSS total score and most other measures of efficacy over the initial 6 to 8 weeks of treatment did not differ between treatments, while improvement at later time points was significantly greater in the SOC group compared with LY2140023. The mean PANSS visit response results were likely impacted by the rate of early discontinuation and the treatment-group difference in discontinuation due to lack of efficacy. A post hoc analysis of only the patients who had PANSS data at the last visit demonstrated similar improvement in the SOC group and the LY2140023 group, although there were significantly more patients with final visit PANSS scores in the SOC group (data not shown). LY2140023 and SOC had comparable negative symptom improvement over the 24 weeks. Further investigation of the efficacy of LY2140023 in the treatment of schizophrenia utilizing placebo-controlled study is warranted.

### Limitations

This study was an open-label study without a placebo treatment arm, preventing definitive conclusions regarding efficacy. This specific design element (that is, open-label) was chosen in order to closely monitor the safety of patients in this initial long-term study. In addition, investigators selected the SOC treatment to be used for each patient in the SOC treatment group, and given the knowledge available for these marketed drugs, SOC treatment may have been optimized by clinicians to a greater extent than for treatment with LY2140023. Normal EEGs were required at baseline, and therefore, any safety results regarding seizures may be limited by this less generalizable population. Similarly, this study focused on moderately ill patients with schizophrenia who had prominent negative symptoms and evidence of functional impairment, and therefore the data are primarily generalizable to patients with a similar symptom profile. Lastly, the overall relatively high discontinuation rate (64%) may limit inference of results on outcome scales.

## Conclusion

This was the first clinical study to examine the long-term safety of a metabotropic glutamate receptor agonist, a potential new therapeutic agent in the treatment of schizophrenia. LY2140023 was comparable to atypical SOC medications in the primary analysis of time-to-discontinuation due to adverse events, a finding demonstrating that LY2140023 was generally well-tolerated over clinically relevant treatment duration. Similar to previous acute studies, LY2140023 had a distinct safety profile characterized by a low incidence of dopamine-related adverse events and a decrease in mean weight compared with SOC treatment.

### Ethics approvals

The following institutional review boards provided ethical approval for the study: Med. Ethikkommission II Fakultät Für Klin. Medizin Mannheim, Comité de Educación Médica, Grupo Ángeles, Centro Para Las Adicciones Y Salud Mental Hidalgo, Hospital Psiquiátrico Estatal, Comité Provinicial de Bioética, CIF-Biotec, Hospital Civil De Guadalajara, Bekhterev Psychoneurological Institute, Mental Health Research Centre of Russian, Serbsky National Research Center, Lipetsk Regional Psychoneurological Hospital #1, National Ethics Committee, Moscow Research Institute of Psychiatry, St. Anthony Hospital Institutional Review Board, Western Institutional Review Board, IU Institutional Review Board.

## Competing interests

DHA, BJK, SB, BAM, IV, SKW are employees and current shareholders of Eli Lilly and Company. DPW is an employee of Collaborative NeuroScience Network, Inc., and served as an investigator on the study.

## Authors’ contributions

DHA and BJK conceived the study, and DHA, BJK, DPW, SB, and IV contributed to the initial design and coordination. SB and BAM led the statistical analyses and interpretation of the data. SKW assisted in writing the initial draft of the manuscript, and coordinated the development of subsequent drafts, including incorporation of revisions to each new version. All authors participated in the interpretation of the data, and revising the manuscript for critically important intellectual content. In addition, all authors read and approved the final version of the manuscript.

## Pre-publication history

The pre-publication history for this paper can be accessed here:

http://www.biomedcentral.com/1471-244X/13/143/prepub
